# Utility of Deep Learning to Address Missing Modalities from Multi-Modal Medical Imaging Studies: A Systematic Review

**DOI:** 10.47852/bonviewaia52026392

**Published:** 2025-10-17

**Authors:** Jinzhao Qian, Ankita Joshi, Hailong Li, Nehal A. Parikh, Jonanthan R. Dillman, Lili He

**Affiliations:** 1 Imaging Research Center, Cincinnati Children’s Hospital Medical Center, USA; 2 Department of Computer Science, University of Cincinnati, USA; 3 Department of Radiology, Cincinnati Children’s Hospital Medical Center, USA; 4 Department of Radiology, University of Cincinnati College of Medicine, USA; 5 Perinatal Institute, Cincinnati Children’s Hospital Medical Center, USA; 6 Department of Pediatrics, University of Cincinnati College of Medicine, USA; 7 Department of Biomedical Engineering, University of Cincinnati College of Medicine, USA; 8 Department of Biomedical Informatics, University of Cincinnati College of Medicine, USA

**Keywords:** deep learning, missing modalities, image synthesis, knowledge transfer, latent space, medical image analysis

## Abstract

Missing modalities pose a significant challenge on multi-modal studies by disrupting the comprehensive analysis of diverse data sources. Deep learning addresses this issue by employing algorithms that can effectively infer and integrate the absent information, thereby ensuring robustness and accuracy of the models while increasing the study’s statistical power. This study aims to provide a systematic literature review on deep learning solutions for missing imaging modalities in multi-modal medical data analysis. Articles on PubMed, IEEE explore digital library, and Scopus were searched in the range from January 2013 to May 2025. This systematic search and review identified 234 articles. Adhering to the specified search criteria, 61 published studies were eligible. Among these, 47% employed image synthesis methods, 20% applied knowledge transfer methods, and 33% used latent feature space-based methods. The paper explores the research gaps and challenges associated within each of these categories. Additionally, this review paper illuminates the popular public datasets for multi-modal studies with missing modalities. Furthermore, it presents evaluation metrics and their key attributes. The review concludes with its limitations and a detailed discussion of current challenges and future directions in this domain.

## 1. Introduction

Over recent decades, medical advances have made it possible to generate abundant medical data for individual patients from various resources (e.g., imaging systems, blood tests, and histopathology data). Medical data obtained from different resources are referred to as multi-modal data. Each data modality represents a different type of data, such as images from medical imaging scans, text from electronic health records (EHR), histopathologic data from tissue specimens, or genetic data [1]. Advanced neural network methods have been applied to interpret these modalities of medical data generated from diverse sources. These methods aim to leverage the complementary information presented in different modalities while also utilizing the unique strengths of each modality to improve model performance and enhance understanding of complex systems [2]. Multi-modal methods have been applied across various clinical domains [3]. Combining multimodal information enables healthcare professionals to develop a holistic view of a patient’s health condition, aiding in diagnosis, treatment planning, and/or therapy monitoring [4]. For instance, in neuroimaging, the integration of structural Magnetic Resonance Imaging (MRI) with functional MRI and diffusion MRI provides detailed information about brain structure, function, and microstructure, enhancing the management of neurological disorders [5]. Similarly, in oncology, combining data from Computed Tomography (CT), MRI, and Positron Emission Tomography (PET) scans improves tumor analysis and treatment response and assessment, enabling personalized cancer care [6]. Furthermore, multi-modal methods drive medical research by exploring complex biological processes, leading to innovative diagnostic and therapeutic advances [2]. Multi-modal data fusion algorithms can fully take advantage of informative and abundant features in cancer survival prediction [7], while multi-modal ensemble techniques are suitable for decision making in medical datasets [8].

One of the most significant challenges to the adoption of multi-modality is the high prevalence of missing data. In real-world clinical settings, it is not uncommon for certain imaging modalities to be unavailable due to equipment limitations, patient-related factors, or technical issues. Additionally, artifacts or inadequate capture of specific regions of interest within an image further complicates the analysis process. Traditional methods for dealing with missing data often entail strategies such as complete case analysis, which assumes that the data are free of missing data and only uses cases with complete datasets for analysis [9], or simplistic imputation techniques such as mean substitution or last observation carried forward [10]. Nevertheless, these methodologies exhibit significant shortcomings. Complete case analysis reduces the study’s statistical power and may lead to biased results if data are not missing at random, while simple imputation methods may introduce inaccuracies and underestimate variability, affecting the reliability of subsequent analyses. The assumption that the dataset has no missing data leads to models only working well theoretically and cannot be adapted to real-world scenarios. Moreover, these approaches fail to fully utilize available information, potentially limiting the insights gained from multi-modal data integration. Whereas, in deep learning-based approaches missing modalities will result in an insufficient dataset for training [11]. This leads to serious problems of decreased model accuracy, potential biases, and unreliable predictions, ultimately compromising the effectiveness of the model in clinical applications.

Deep learning methods have provided an alternative solution to the missing modality problem [12]. Their inherent capability to discern intricate patterns in data renders it highly suitable for managing the complexities of high-dimensional and heterogeneous multiple modalities, eliminating the need for manual feature engineering [13]. They can automatically extract and integrate relevant features from multimodal MRI, CT, and PET scans for accurate disease diagnosis without the need for manual feature extraction [14]. Besides, their adaptability ensures precise imputation of missing data, with the utilization of extensive datasets bolstering generalization across varied clinical contexts. For example, in the EHR, deep learning models exhibit proficiency in facilitating more accurate imputation by effectively learning intricate patterns and correlations within the patients’ data, which enables precise predictions and the customization of treatments to the unique needs of individual patients [15]. Furthermore, they adeptly incorporate contextual cues and spatial relationships within multiple modalities, enhancing data fidelity and facilitating thorough analyses. For instance, in pathology slides, deep learning models can simultaneously analyze histopathological images alongside clinical data and genetic information to deliver a thorough understanding of disease progression and treatment response [16].

The past few years have witnessed heightened focus on utilizing deep learning methods to address the issue of missing data in multi-modal MRI-based analysis, with published surveys discussing the missing modality issue of MRI data [17, 18]. However, these surveys typically group methods based on technical implementation details or specific modalities and mainly focus on the brain tumor segmentation task, which makes cross-task generalization less clear. By contrast, our framework emphasizes the underlying strategy for handling missing modalities, providing a broader and more unifying perspective. Therefore, in this work, we provide a systematic literature review focusing on all types of imaging modalities and applicable to all tasks within the medical domain like image quality analysis, classification, prediction, and segmentation. Such a review will furnish invaluable insights into the available solutions, limitations, and optimal practices associated with deploying deep learning strategies for addressing missing data issues in multi-modal medical data analysis. In this review, we summarize the deep learning-based methods which address the missing modality problem. In that, we target methods that specifically provide solutions for missing images. We aim to cover every contributory method in the last decade. This survey contributes to the following:

1. Systematically review missing modality deep learning techniques in the last 10 years which have provided solutions to missing modality issues in the context of multi-modal medical image analysis.
2. Categorize the identified solutions based on their approach and present their benefits and limitations.
3. Summarize datasets and assessment criteria applied in the studies.
4. Analyze the main findings and outline future directions for research in this domain.

The remainder of the review is organized as follows: Section 2 outlines the review planning; Section 3 presents key findings; Section 4 discusses and interprets the results; Section 5 states limitations in our research; Section 6 highlights future directions; and Section 7 makes the conclusion of the review.

## 2. Review Planning

This section focuses on organizing the review: clearly specifying the research questions relevant to the study, detailing the information sources, and outlining the inclusion criteria.

### 2.1. Key research questions

1. What approach is proposed to address the missing modality problem?
2. Which dataset is used?
3. Under which methodological category does the proposed approach fall?
4. What pre-processing approaches are used?
5. What criteria are used for evaluation?
6. Is the code publicly available?
7. Does the article carry out external validation?

### 2.2. Data sources and search strategy

Our study followed the Preferred Reporting Items for Systematic Reviews and Meta-Analyses (PRISMA) guidelines [19]. We searched both general databases (Scopus, PubMed) and a subject-specific database (IEEE Xplore) to ensure broad biomedical coverage while also capturing technical contributions often published in engineering venues. The search strategy was developed for the purpose of reviewing deep learning methods for missing modalities in multimodal medical imaging. Queries combined terms related to multimodality, missing data, clinical tasks (e.g., classification and segmentation), medical imaging context, and deep learning techniques. This blended approach allowed us to balance breadth and depth and ensured that both methodological and application-oriented studies were included. The queries used for the respective sources of publications spanning from January 2013 to May 2025 have been listed in Table 1.

### 2.3. Inclusion criteria

Inclusion requirements were: (a) original research article; (b) published within January 2013 and May 2025; (c) published in English language; (d) required to be a multi-modal study involving different modalities of medical images, or imaging and any other non-imaging modality; (e) proposing solutions to missing imaging modality; (f) proposing deep learning-based solutions; and (g) applied to any classification, prediction, or segmentation task in medical domain.

**Figure 1:**
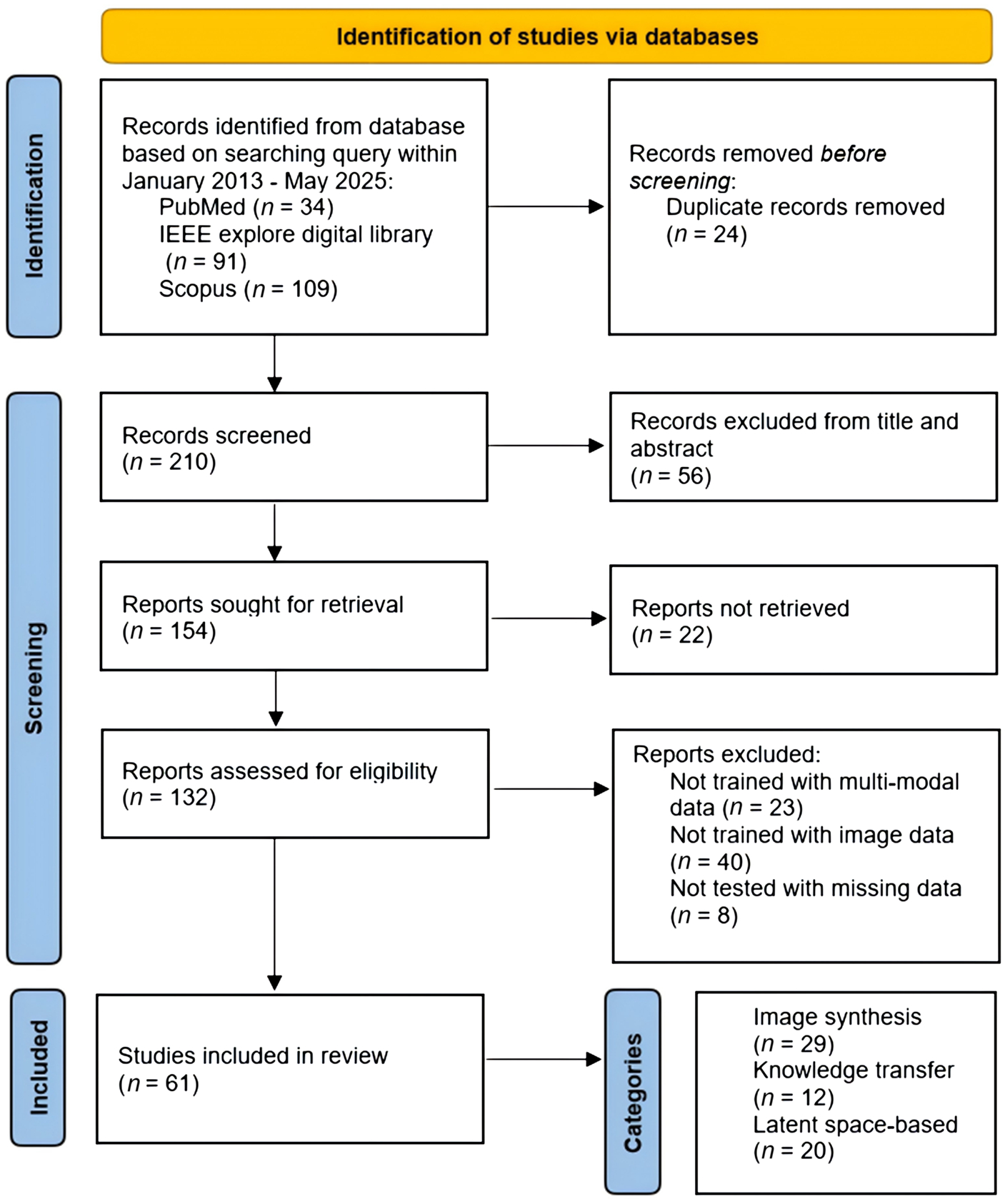
Flow diagram for retrieved articles based on the PRISMA guidelines

Specifically, papers’ titles and abstracts were sought on each of the previously mentioned information sources using the respective search queries as given in Table 1. Figure 1 presents the flow diagram based on the PRISMA guidelines. In total, 234 records were initially identified. Following the first exclusion phase, 210 remained for abstract screening. Of these, 132 met the criteria for full-text review. Finally, 61 studies were retained for this review, with 29 image synthesis methods, 12 knowledge transfer methods, and 20 latent feature space-based methods. The resulting 61 studies were cross-checked by a second reviewer, and disagreements were resolved through team discussion until consensus was reached.

### 2.4. Data extraction

For each included study, we extracted key information using a structured template, covering publication year, dataset(s), imaging modalities, methodological approach, preprocessing steps, model architecture, evaluation metrics, performance results, code availability, and use of external validation. Data extraction was carried out by one reviewer and verified by a second. To synthesize findings, studies were grouped into three methodological categories (image synthesis, knowledge transfer, and latent feature space–based methods). Within each category, results were compared in terms of performance, robustness to missing modalities, reproducibility (code availability, external validation), and task type. Evidence was summarized narratively and with structured tables (Tables 4–6), alongside a standardized comparison table (Table 8) highlighting core attributes such as performance, robustness, and reproducibility.

## 3. Results

With above search strategy, we retrieved 234 articles published between January 2013 and May 2025. After manually removing articles that did not meet the inclusion criteria and searching the bibliography of eligible articles, 61 articles met the inclusion criteria. The results are presented as follows: First, we outline the datasets utilized across the articles included in this review. Then, we present a compilation of the 61 research articles, organized based on the established categories and finally we summarize the evaluation metrics used by these studies.

### 3.1. Dataset

We list all the public multimodal datasets used by the 61 articles included in this review in Table 2. For each dataset we include the online location to access the dataset, the dataset details in terms of number of subjects, and the modalities available with the dataset.

### 3.2. Deep learning methods with missing imaging modality

The purpose of this section is to investigate the questions raised in the review planning process in Section 2. Without considering specific tasks (e.g., classification, regression, or segmentation), multi-modal deep learning models take multiple modalities of imaging data, and extract the high-level feature representations (i.e., feature embeddings).

**Figure 2:**
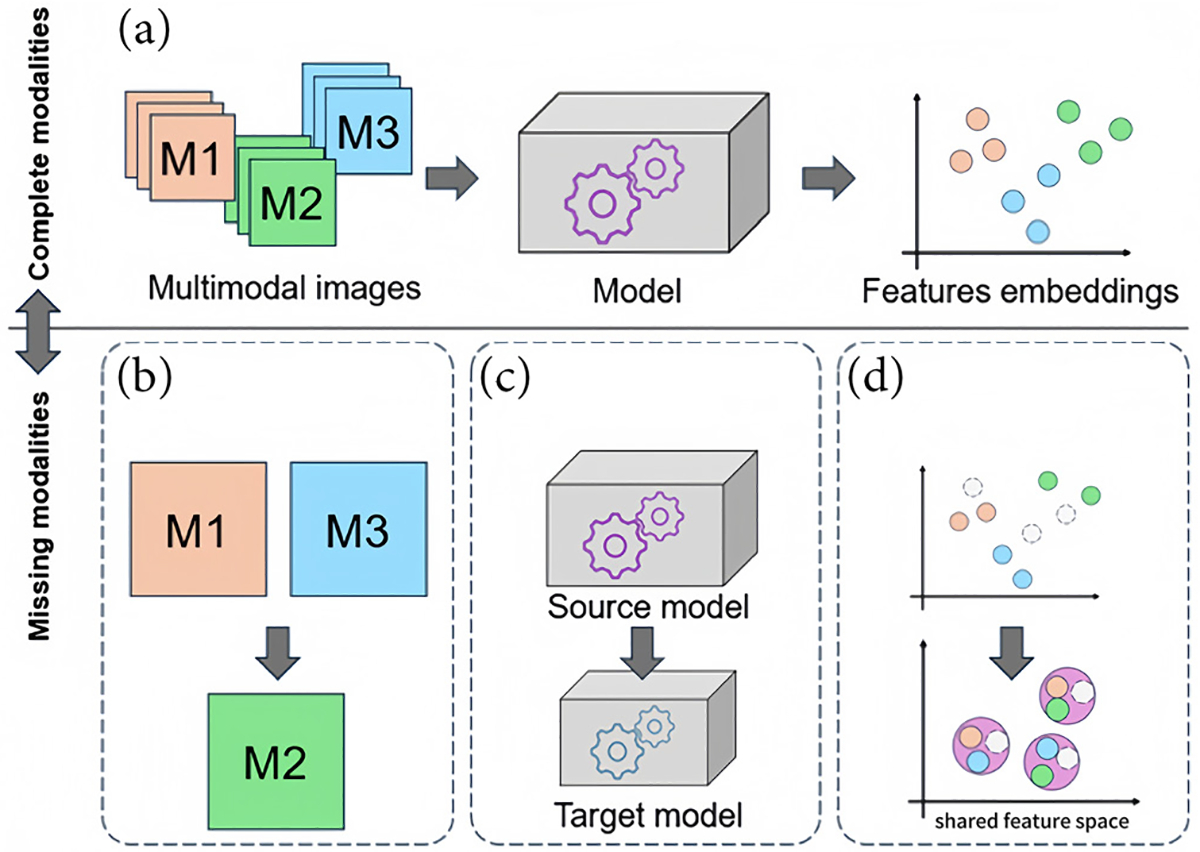
Illustration of methods by category. (a) With complete modalities, multimodal images are directly used for feature extraction. When modalities are missing, three strategies are applied: (b) synthesis methods generate absent modalities from available ones, (c) knowledge transfer leverages models trained on complete data to guide models trained on partial data, (d) latent space methods fuse embeddings into shared representations

**Figure 3:**
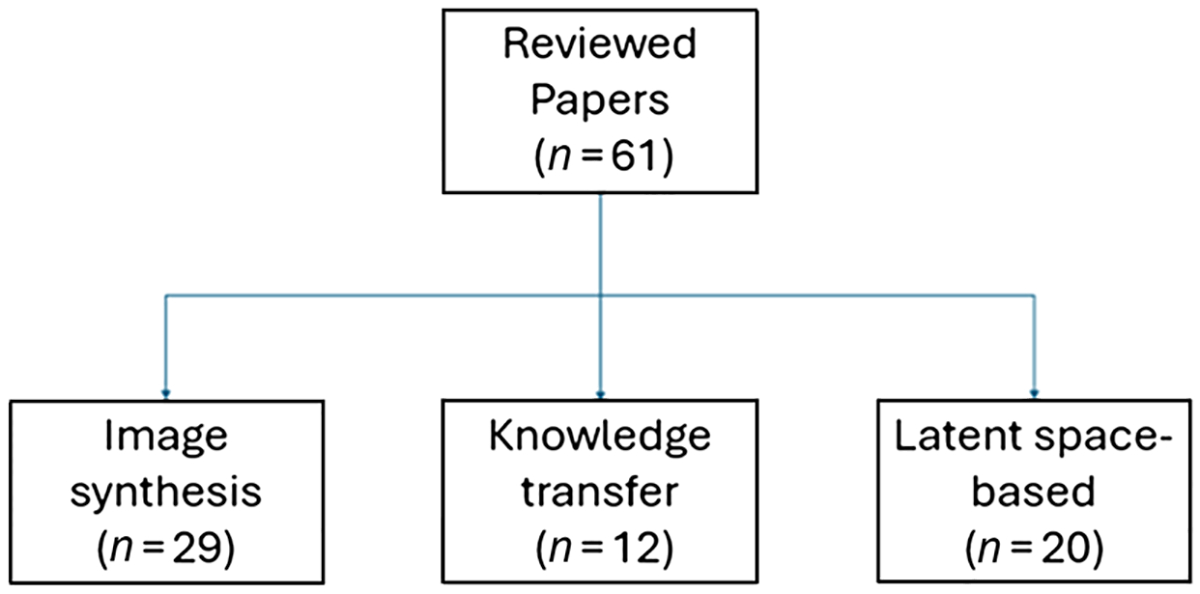
Taxonomy diagram for retrieved articles in this study

In this work, we categorized all reviewed deep learning methods into (i) image synthesis methods, (ii) knowledge transfer methods, and (iii) latent feature space-based methods. Figure 2 illustrates the categories presented in this review. Briefly, image synthesis methods synthesize the missing imaging modalities with existing ones, knowledge transfer methods focus on establishing new architectures/deep learning models to transfer knowledge, and latent feature space-based methods work in the shared feature sub-space. We present all 61 articles identified in this review based on these three categories. Table 3 presents all identified papers under each category, with 29 studies in image synthesis methods, 12 studies in knowledge transfer methods and 20 studies in latent feature space-based methods. Figure 3 summarizes their distribution in a taxonomy diagram. In Tables 4–6, for each category of methods, we provided the preprocessing pipeline of the method, the basic architecture of the deep learning model, the main context of the proposed solution for handling missing modality, the medical task performed, and the evaluation metrics used to assess the performances. To show the accessibility of the methods, we also mention code availability of the proposed solution and the use (or absence) of external validation.

#### 3.2.1. Image synthesis methods

Image synthesis methods aim at recovering the missing imaging modalities from the available imaging modalities. These methods typically were developed using Generative Adversarial Networks (GAN) [97] and its variants [98]. The synthesis models consist of two subnetworks: one generator that synthesizes the missing modalities from available ones, and one discriminator that judges whether the input image is real or synthesized by generator [99]. These subnetworks are trained simultaneously under an adversarial loss. As a result, the generator’s performance is improved to create satisfying images as compensation to missing ones. To improve the quality of synthesized image, multi-modal learning techniques are applied in the generator to correlate features from multiple modalities [40]. The synthesized images can either serve as supplementary training data to help the downstream models better understand the tasks or be used to augment training data, in order to improve the models’ robustness and generalization capabilities.

Despite the obvious advantage of recovering missing imaging modality information directly, the training cost of GAN is relatively high due to their model complexity. What is more, the training of GAN will become unstable when encountering issues like mode collapse [100], resulting in poor synthesis performance. Table 4 gives a detailed list of all studies included in this review that use image synthesis approaches.

#### 3.2.2. Knowledge transfer methods

Knowledge transfer methods first develop a source model that encodes all data modalities. The representations learned from the source model are then transferred to the target model, which has incomplete modalities as input. This target model is then utilized for the downstream task [68]. There are two ways to transfer the learned modalities from the source model to the target model, namely, using the knowledge distillation method [65], or by using domain adaptation [66].

Knowledge distillation involves training the target model in accordance with the source model. First, the source model with a larger number of parameters is trained with the full modalities until it achieves high performance on the target task. The target model of less complexity learns from the source model by capturing the encoded knowledge in its outputs and then is trained with incomplete datasets. The forward propagation is performed on both models while the back propagation is only performed on the target model. The output of the source model can be used as soft labels to guide the training of target model [103]. The discrepancy between the target model’s predictions and the soft labels of the source model is measured by the target’s distillation loss, which is combined with traditional learning loss in the training of the target model.

Domain adaptation methods are typically used in transfer learning approaches for handling missing modalities to uncover the common latent features across the source and target domains. The source model encodes complete modalities, whereas the target model encodes the incomplete modalities separately, and features are extracted from both models. A discriminator is used to bridge the domain gap between the output of the source and target model. A similarity loss is used with the discriminator to map the features to a similar distribution, and a consistency loss is applied to minimize the distance between the distributions [104].

Knowledge transfer methods outperform other non-synthesizing methods in multiple conditions of missing modalities and are capable of transferring to other tasks [67]. Nevertheless, it could be affected by limited availability or imbalanced datasets, resulting in difficulty in training or a decrease in performance [71]. Table 5 gives a comprehensive list of all the studies covered in this review that use knowledge transfer approaches.

#### 3.2.3. Latent feature space-based methods

Latent feature space-based methods offer a typical solution to handling missing modalities by focusing on deriving meaningful features from existing modalities alone, bypassing intermediate processes such as imputation [106] or synthesis [107]. Latent feature space-based methods involve independently learning and embedding of the input image into a latent space for each modality. Each of the modalities is encoded respectively to extract its specific feature representations. Subsequently, these modalities are translated into a shared latent space. Fusion strategies such as mean and variance operations are utilized to establish one common latent embedding for all modalities, so that missing features can be recovered [96]. Once the shared representation is obtained, a decoder is used to reconstruct the data from it to perform the downstream tasks. Such methods are robust to missing imaging modality by learning a shared representation of the available modalities [81].

However, projecting data into a latent space can lead to information loss from the original modalities. This loss can affect the overall performance of the model, particularly if the missing modalities contain critical information [78]. Also, the learned latent vectors of individual modalities from independent encoders are fused to create a shared representation. However, this assumes that each modality is equally informative and contributes equally to the final task, which may not always be true. Besides, interpreting the results of models based on latent feature space methods can be challenging, especially when the relationships between modalities are complex or non-linear. Table 6 gives a detailed list of all the studies included in this review that use latent feature space-based approaches.

### 3.3. Evaluation metrics

In this section, we review the evaluation metrics applied for validating the performances of the methods in missing modalities scenarios. In classification tasks, accuracy, sensitivity, specificity, and area under the curve (AUC) are the most popular choices for assessment. Dice similarity coefficient (DSC) is widely used for measuring the segmentation results, while peak signal-to-noise ratio (PSNR) and structural similarity index measure (SSIM) intuitively display the quality of synthesized images of missing modalities. Table 7 gives a list of all the evaluation metrics used in the articles included in this review with the following attributes for each: the name and abbreviation of the metric, the formulation to compute the metric, and the usage of the metric.

## 4. Discussion

This paper offers a systematic review of deep learning methods dealing with missing imaging modalities in medical analysis in terms of the most popular public datasets used, the different types of deep learning approaches developed, and the most common metrics for evaluating the performance of the approaches. Though prior works have reviewed deep learning approaches for brain tumor segmentation tasks under the condition of missing MRI modalities, a systematic review that includes deep learning methods under the circumstance of missing medical imaging modalities regardless of the tasks to be performed, has not been published yet. We identified 61 papers in this review that focus on deep learning-based solutions that we categorized into image synthesis methods, knowledge transfer methods, and latent feature space-based methods. Unlike bibliometric systematic reviews, which primarily analyze publication volume, author networks, or keyword co-occurrence, our review uncovers trends at the methodological level. These include shifts in the types of deep learning models employed (e.g., increasing use of U-Nets and transformers), the prevalence of specific evaluation metrics across tasks (e.g., SSIM for image synthesis, DSC for segmentation, and AUC for classification), and reproducibility practices (e.g., proportion of studies with external validation or public code). We therefore provide a systematic methodological synthesis rather than a bibliometric mapping, which we believe is more directly useful to the medical imaging community.

In the last decade, neural network approaches have gradually been recognized for their capabilities of tackling missing modalities in medical analysis. Overall, we find that both latent feature space-based methods favored for their straightforward deployment, and image synthesis methods due to the popularity of GAN, are the more favorable techniques for dealing with missing modalities. Knowledge transfer methods show noteworthy performance in cases where multiple modalities of the data are missing.

Firstly, our results show that 47% of the reviewed papers use image synthesis methods. 17% of articles focus on methods that are implemented for the segmentation purpose [44, 46, 55, 56, 64], 38% are implemented for the classification tasks [37, 39–41, 47, 50, 53, 57–59, 63], and 4% for the prediction use [48]. 41% of the reviewed papers under image synthesis-based methods focus on the evaluation of the quality of synthesized images [36, 38, 42, 43, 45, 49, 51, 52, 54, 60–62]. All the reviewed image synthesis methods utilize GAN or its extension framework, consisting of one or two generators and one discriminator. However, the architecture of the generator to synthesize images for the recovery of the missing modalities varies. Nearly half of the GAN-based synthesis networks apply CNN-based structure as the generator. Nevertheless, CNN-based generator has several drawbacks, such as fixed input–output channels, lack of interpretability [45], and loss of low-level spatial information [49]. U-net, which is capable of channel-wise feature fusion, wins more favor in the choice of generator. It allows region-specific feedback, enhancing the generator’s learning process and leading to superior image synthesis quality. According to our review, U-net is utilized as the generator in 36% of the papers and has been especially popular in the last three years. It is worth mentioning that 12% of the papers combine CNN or U-net with the transformer in the image synthesis network. Leveraging the attention mechanism, the transformer performs efficient and accurate synthesis by capturing cross-modal correlations within input modalities while removing redundant information, and the interpretability of the model could be increased by visualizing the attention score. In [44], an efficient generator is designed by the combination of transformer and CNN, enabling the model to have global sensitivity as well as detailed local modeling. In terms of the discriminator, almost all the papers choose CNN. Only a few papers attempt to make effective improvements on the discriminator, such as the task-induced design of the discriminator aimed at integrating image synthesis with downstream tasks [53].

Next, 20% of the reviewed papers use knowledge transfer methods, which have garnered increased attention among researchers in recent years. 58% of these methods are implemented to perform segmentation tasks [65, 71–76], and 42% are performed on classification tasks [66–70]. All the methods for segmentation purpose choose U-net, while for classification the choice of network ranges from CNNs to GAN. The difficulty of designing the network, the high training cost of the source model as well as the interpretability of the model have always been the major concern concerns for knowledge transfer methods [119]. Even though none of the reviewed papers completely overcame these hindrances, some of them made notable progress. In [71] authors disentangled features on the modality level and employed a contrastive learning-based learning scheme in the spatial and frequency domain to exploit more explicit relations between modalities. Similarly in [66], a contrastive learning-based loss was applied to direct the optimization of the target model from the source model. The authors in [68] combined image synthesis models with domain adaptation by implementing two GAN to generate missing modalities and transfer knowledge within the learned feature space.

Thirdly, our results show that 33% of the reviewed papers use latent feature space-based methods. Among them, 55% are implemented for the purpose of segmentation [77, 81, 84, 86–89, 92–94, 96], 20% are implemented for classification tasks [79, 80, 85, 90], and the rest are implemented for various uses, such as prediction [78, 82, 83], missing modality imputation [91] and synthesized image evaluation [95]. Of the reviewed latent feature space-based methods, 80% rely on convolution neural networks (CNNs) to extract latent features. Specifically, around 50% of them use U-net, which has a comprehensible structure and performs well on the segmentation tasks [120]. Notably 10% of the reviewed papers leveraged transformers to achieve satisfactory outcomes [79, 84]. Considering that the bias of convolution limits the ability to harness cross-modal relationships [86], a multi-modal transformer can model the correlated high-level features from different modalities by the attention mechanism [110]. Moreover, the transformer has inspired researchers to combine the attention mechanism with the traditional encoder–decoder model, enabling the network to highlight important latent features while suppressing irrelevant ones [77]. The remaining 10% of the methods use RNN-based blocks in their network when dealing with longitudinal missing modality [78, 82]. Multi-modal data fusion strategies are utilized by most latent feature space-based methods to combine the multi-modal extracted features into latent space representation. They contribute to exploiting the latent feature space and emphasizing the major features from multiple modalities. The fusion strategies based on arithmetic operations such as merging the features by computing mean and variance are simple but effective when the purpose is to reinforce the model’s robustness. More complex fusion strategies, including L1 or L2 distance minimization [95] and attention-based fusion [89], have also been proposed. However, it is difficult to decide which strategy is best for handling multi-modal missing imaging modality since none of them assure the model to learn a shared latent representation from different intensity distributions of different modalities. Interpretability of the multi-modal deep learning models is another concern for their further applications in medical image analysis [121]. Consequently, researchers are striving to enhance the interpretability of their models. For example, in [81] authors not only presented a latent feature space-based U-net effective for both classification and segmentation tasks but also enhanced the interpretability via the t-SNE visualization of the latent feature space.

Although a formal meta-analysis was not feasible due to the substantial heterogeneity across studies in terms of datasets, tasks, and performance metrics, in Table 8, we provide a quantitative summary of the three methodological categories, comparing their typical applications, reported performance, robustness to missing modalities, external validation, and code availability. Across the three categories, reported performance is generally similar. Segmentation tasks, which are mostly evaluated on the BraTS dataset, show DSC values around 0.86–0.88, while classification tasks on the ADNI dataset reach AUC values of about 0.83–0.85. For image synthesis, SSIM is typically around 0.93. The main differences lie in robustness and research practice: image synthesis methods are usually tested on specific missing modalities, show more frequent external validation, but have the lowest code availability. In contrast, knowledge transfer and latent space methods are more often tested in missing modalities scenarios, though they are less often externally validated, with code availability being high (~70%) and moderate (~30%), respectively.

While the above categories provide a useful framework to organize the literature, they are not fully separate. Some studies use methods that overlap across categories. For example, certain works generate synthetic images to expand datasets while also applying knowledge transfer to improve model performance [68]. Others combine latent feature space analysis with image synthesis to strengthen prediction tasks [36]. These mixed approaches show that methods in this field are often built on each other, and that combining techniques from different categories can be especially useful for tackling the challenges of multimodal medical imaging.

In the review, we have summarized the datasets used for missing modality studies. Regarding the most popular datasets used to develop missing modalities techniques, 60% of the approaches included in this review used the BraTS dataset and 29% selected the ADNI dataset for training. Most datasets contain multiple MRI modalities, resulting in similar preprocessing pipelines. It is worth noting that public datasets partly reflect real-world clinical practice. They are derived from real patients and usually include preprocessing steps such as normalization or artifact reduction, which are also common in practice. This makes them useful for benchmarking and method development. However, they are not fully representative, as the released data are often high-quality and relatively clean. Cases with noise, missing data, or poor annotations are usually filtered out. Therefore, results based only on public datasets may not fully capture the challenges of everyday clinical scenarios.

With respect to experimental design, preprocessing was the most frequently discussed aspect. For MRI preprocessing, normalization, registration, and skull stripping are the top three frequently used methods, while for other modalities such as PET, registration and normalization remained standard practice. In terms of computational resources, 90% of the papers listed in this review used Graphical Processing Units (GPUs) for their training. Only 25% of the reviewed papers provided a public code, half of which are latent feature space-based methods, promoting reproducibility and collaboration for advancing studies. There are 42% reviewed papers using multiple datasets, 23% of which perform an external validation [38, 40, 50, 52, 55, 59], which helps to identify biases or variability present in the training samples while strengthening the robustness and dependability of their models.

Lastly, a detailed summary of all evaluation metrics used by the papers included in this review is provided in Section 3.3. In most studies, the chosen assessment criteria depend on the task categories. Around 34% of papers included in this review evaluate their methods under one missing modality condition. Among the rest of papers tested on multiple missing modalities scenario, half of them carry out ‘robustness evaluation’ [122], i.e. assessing their networks on every possible combination of missing modalities. For non-synthesis methods, it is difficult to quantify how well the recovery is because in some categories, it is not even possible to verify the quality of the recovered modality. For image synthesis methods, such evaluation is available due to the presence of ground truth. However, it is hard to compare the recovered modalities within different image synthesis models even if they are applied to the same task and the same dataset, if they synthesize different imaging modalities. Also, since most image synthesis-based methods use CNN networks that rely on fixed channels of input and output, it is difficult for them to perform a robust evaluation as a separate model will be required for each possible input–output scenario [45]. In contrast, nearly half of the articles on latent feature space-based methods and knowledge transfer methods implement the ‘robustness evaluation’.

## 5. Limitation

Firstly, the review was conducted by only two reviewers. Although cross-checking was carried out, the small number of reviewers may have introduced some subjectivity in study selection and data extraction. Secondly, the included studies are more weighted toward computer science and methodological aspects, while clinical perspectives may be underrepresented. This could limit the assessment of clinical relevance. Thirdly, it was difficult to compare results across studies in a quantitative way, since they used different datasets, preprocessing steps, evaluation metrics, and tasks. The lack of standardized benchmarks makes direct comparison challenging. Nevertheless, given the systematic search across multiple major databases and the structured synthesis process, we are confident that this review covered the most relevant studies and that the findings capture the current landscape of research on deep learning methods for missing modalities in multimodal medical imaging.

## 6. Future Direction

Despite the rapid development of deep learning methods, there is still a long way to go to solve the missing modalities problem perfectly. In this section, we will discuss the limitations of current approaches and indicate the possible solutions concerning them.

A major challenge for adopting deep learning-based models in a clinical-setting is posed by that lack of model interpretability. For clinical decision-making applications, model interpretability and introspection are crucial components. It is known that features extracted from deep learning models are abstract and may not always be clinically relevant [6]. Such models will not generalize well on unseen data or be biased against certain populations. However, the interpretation of deep learning models is an active research topic and multiple methods have been proposed [123–127] that allow us to visualize which parts of the data the model considers important for its predictions, even though the underlying feature representations remain abstract. Ref. [123] shows a popular deep learning technique that visually identifies the key areas within an input image influencing the model’s predictions. It is beneficial in shedding light on how decisions are made by multi-modal deep learning models applied to medical images. Emerging solutions aim not only to enhance interpretability but also to uncover associations between different modalities [6, 128].

From the perspective of multi-modal data dimensionality, we observe in our results that 2D is a more popular choice than 3D. Although 3D medical image analysis offers advantages in terms of capturing richer spatial information and potentially improving prediction accuracy, it comes with the increased complexity of models. For instance, CNNs used for 3D image analysis may require deeper architecture or incorporate additional layers to handle the data. Adapting transfer learning is a potential solution to this challenge. Pre-trained models from related domains will help the 3D target model to initialize more efficiently and reduce the need for extensive labeled data [129]. Another noteworthy approach is to integrate attention mechanisms into 3D models. By focusing on the important parts and ignoring irrelevant regions of the 3D images, the attention modules can reduce the computation cost and improve the interpretability of the 3D models [130].

Another important future direction for successfully dealing with missing modalities will be gaining data diversity. The BraTS and the ADNI datasets provide a large sum of image data for the reviewed papers’ experiment; however, most of the data are of MRI modalities, bringing inadequate data diversity issues. As it is difficult to collect various and complete imaging data from clinical scenarios, generated images might be a better way to enrich the data diversity. To achieve such a purpose, more research on the fidelity of the synthesized images and the interpretability of image synthesis methods are required. Other modalities of data such as clinical records or genomic information are also applicable to expand the data variety and improve the effectiveness of the models.

In addition to data diversity, another critical consideration is the lack of consistent ways to evaluate methods across studies. While various papers report quantitative metrics, differences in tasks, datasets, and evaluation metrics make it hard to compare results directly. Therefore, future work should create standardized benchmarks and shared evaluation protocols. This would make cross-study comparisons more meaningful, improve reproducibility, and provide reliable baselines to guide future research.

Despite the popularity of deep learning methods, a major roadblock for its widespread adoption is the unavailability of large-scale groundtruth datasets with all multimodal data. This challenge arises primarily from the labor-intensive process of manual annotation, privacy concerns in the clinical domain, and biases introduced by the typically small size of patient cohorts, which often represent high socioeconomic status [131, 132]. Popular approaches like data augmentation [133], semi-supervised learning [134], transfer learning [135], and automated annotation [136] offer promising solutions to address the problem of insufficient labeled data.

## 7. Conclusion

This review identified and summarized 61 relevant papers through the search process in accordance with the PRISMA guidelines. Our research is distinct in its focus on systematically reviewing multi-modal deep learning methods developed over the last decade dealing with missing imaging modality issues in medical image analysis. Central to our investigation are key research inquiries answered in the results section, illustrating the methodologies employed in medical image analysis with missing modality, cataloging publicly available datasets for researchers, and encapsulating prevalent evaluation techniques. We provide a detailed discussion regarding our findings and identify notable research gaps and their potential solutions. The literature explores addressed questions and analyzes them in the following sections, highlighting a rapidly growing and globally significant field of interest.

## Figures and Tables

**Table 1:** Search query

Source of information	Query used
PubMed^1^	((multimodal) OR (fusion) OR (ensemble) OR (feature integration) OR (multiparametric)) AND ((missing data[Title/Abstract]) OR (missing modality[Title/Abstract]) OR (incomplete data[Title/Abstract])) AND ((classification [Title/Abstract]) OR (regression[Title/Abstract]) OR (prediction[Title/Abstract]) OR (diagnosis[Title/Abstract]) OR (segmentation[Title/Abstract])) AND ((medical image) OR (medical application)) AND (deep learning)
IEEE explore digital library^2^	
Scopus^3^	(ALL (multimodal) OR ALL (fusion) OR ALL (ensemble) OR ALL (feature_integration) OR ALL (multiparametric)) AND (TITLE-ABS-KEY (missing AND data) OR TITLE-ABS-KEY (missing AND modality) OR TITLE-ABS-KEY (incomplete AND data)) AND (TITLE-ABS-KEY (classification) OR TITLE-ABS-KEY (regression) OR TITLE-ABS-KEY (prediction) OR TITLE-ABS-KEY (diagnosis) OR TITLE-ABS-KEY (segmentation)) AND (ALL (medical_image) OR ALL (medical_application)) AND (ALL (deep_learning) AND (LIMIT-TO (SUBJAREA,“COMP”)) AND (LIMIT-TO (DOCTYPE,“ar”) OR LIMIT-TO (DOCTYPE,“cp”))

1 (https://pubmed.ncbi.nlm.nih.gov/)

2 (https://ieeexplore.ieee.org/Xplore/home.jsp)

3 (https://www.scopus.com/)

**Table 2:** Summary of the datasets used in the papers included in the review

Multi-modal datasets			
Dataset	Online link	Subjects	Modalities
Alzheimer’s Disease NeuroImaging Initiative (ADNI) [20]	https://adni.loni.usc.edu/	200 elderly cognitively normal, 400 Mild Cognitive Impairment (MCI), and 200 Alzheimer’s disease (AD) subjects	Clinical, genetic, MRI, PET, biospecimen
Brain Tumor Segmentation Challenge (BraTS (2021)) [21]	http://braintumorsegmentation.org/	2040 glioma cases (8000 Multiparametric MRI scans)	MRI modalities: T1, T1 post-contrast (T1c), T2, T2 Fluid-Attenuated Inversion Recovery (FLAIR)
Ischemic Stroke Lesion Segmentation (ISLES) [22]	https://www.smir.ch/ISLES/Start2015	36 cases on subtask: sub-acute ischemic stroke lesion segmentation (SISS)	MRI modalities: T1, T1c, T2, T2 FLAIR, Diffusion-weighted Imaging (DWI)
IXI Brain Development	https://brain-development.org/ixi-dataset/	Nearly 600 scans from normal, healthy subjects	MRI modalities: T1, T2, Proton Density (PD), Magnetic Resonance Angiography (MRA), DWI
Open Access Series of Imaging Studies (OASIS-3) [23]	https://sites.wustl.edu/oasisbrains/home/oasis-3/	1378 participants collected across several ongoing projects through the WUSTL Knight ADRC over the course of 30years, include 755 cognitively normal adults and 622 individuals at various stages of cognitive decline ranging in age from 42–95yrs.	MRI modalities: T1, T2, T2FLAIR, etc. PET, CT
The Cancer Genome Atlas Program (TCGA)	https://www.cancer.gov/ccg/research/genome-sequencing/tcga	Including Kidney Clear Cell Carcinoma (KIRC) (385 cases), Liver Hepatocellular Carcinoma (LIHC) (287 cases), Esophageal Carcinoma (ESCA) (153 cases), Lung Squamous Cell Carcinoma (LUSC) (438 cases), Lung Adenocarcinoma (LUAD) (452 cases), and Uterine Corpus Endometrial Carcinoma (UCEC) (387 cases), etc.	Genomic data, MRI, Region Of Interest (ROI) images, diagnostic Whole Slide Imaging (WSI) images, clinical records, etc.
MIMIC Chest X-ray (MIMIC-CXR) [24]	https://physionet.org/content/mimic-cxr/2.0.0/	65,379 patients presenting to the Beth Israel Deaconess Medical Center Emergency Department between 2011–2016	Clinical time series data, X-ray
Parkinson’s Progression Markers Initiative (PPMI) [25]	https://www.ppmi-info.org/access-data-specimens/download-data/	400 recently diagnosed Parkinson Disease and 200 healthy subjects	T1 MRI, Diffusion Tensor Imaging (DTI) images, Single Nucleotide Polymorphism (SNP)
LONI Probabilistic Brain Atlas (LPBA40) [26]	https://loni.usc.edu/research/atlases	40 healthy, normal subjects	T1 MRI
Synapse [27]	https://www.synapse.org/#!Synapse:syn3193805/wiki/217753	34 subjects for MRI and 30 subjects for CT	MRI, CT
Combined (CT-MR) Healthy Abdominal Organ Segmentation (CHAOS) [28]	https://chaos.grand-challenge.org/	80 patients, 40 of them (22 males, 18 females, ages between 18 and 63) went through a single CT scan and 40 of them (23 male, 17 female, ages between 18 and 76) went through MR scans	MRI, CT
Chinese Brain Molecular and Functional Mapping (CBMFM) [29]	Upon request	646 subjects from 18 to 82 years old collected from four medical centers	MRI modalities: T1, T2, T2FLAIR
Early life adversity biological embedding (eLABE)	Upon request	127 neonates (postmenstrual age=41.1 ± 1.5 weeks, female N = 59, white N = 42)	MRI modalities: T1, T2, resting state functional MRI (rs-fMRI)
Environmental influences on child health outcomes (ECHO) [30]	Upon request	including 10 infants (age=41.2 ± 1.9 weeks, female N = 5, white N = 8)	MRI modalities: T1, T2, rs-fMRI
Multiple Sclerosis with the MS Grand Challenge (MSGC) [31]	http://www.ia.unc.edu/MSseg/	45 cases, 25 from the Boston Children’s Hospital (CHB) and 20 from the University of North Carolina (UNC)	MRI modalities: T1, T2, T2FLAIR
Relapsing Remitting Multiple Sclerosis (RRMS)	Upon request	300 RRMS patients (mean age = 37.5, surface distance (SD) = 10.0)	MRI modalities: T1, T2, T2FLAIR, T1c
Chinese Longitudinal Aging Study (CLAS) [32]	Upon request	1068 elderly Chinese (42.2% male), mean age of 72.8 years (SD = 8.5) completed a comprehensive cognitive, psychosocial and mental health assessment	T1 MRI
The Australian Imaging, Biomarkers and Lifestyle (AIBL) [33]	Upon request	1112 subjects, 211 AD, 133 MCI, 768 healthy controls	T1 MRI, PET
UNC/UMN Baby Connectome Project [34]	Upon request	500 typically developing infants, toddlers, and preschool-aged children between birth and 5 years of age	MRI modalities: T1, T2, DWI, rs-fMRI
MIDAS [35]	Upon request	34 healthy subjects, ranging in age from 19 to 72 and of both sexes, 27 tumor cases included thirty lesions	MRI modalities: T1, T2, MRA

**Table 3:** Methodological categories identified in this review and their corresponding studies

Category	References	Total number
Image synthesis methods	[36], [37], [38], [39], [40], [41], [42], [43], [44], [45], [46], [47], [48], [49], [50], [51], [52], [53], [54], [55], [56], [57], [58], [59], [60], [61], [62], [63], [64]	29
Knowledge transfer methods	[65], [66], [67], [68], [69], [70], [71], [72], [73], [74], [75], [76]	12
Latent space-based methods	[77], [78], [79], [80], [81], [82], [83], [84], [85], [86], [87], [88], [89], [90], [91], [92], [93], [94], [95], [96]	20

**Table 4:** Image synthesis methods dealing with missing imaging modality

Image synthesis methods								
Year/author/citation	Dataset/no. of subjects/modalities	Main context	Architecture	Preprocessing	Task	Metrics/performance	Code	External validation
2025/Kebaili, et al. [36]	BraTS 2021/1251 (60% training, 20% validation, 20% testing)/MRI (T1, FLAIR, T1c and T2)	Propose a diffusion-based generative model that studies a unified feature representation over full input modalities	Variational autoencoders	Resizing	① synthesized image quality measurement	Mean squared error (MSE); Peak signal-to-noise ratio (PSNR); SSIM; Learned perceptual image patch similarity (LPIPS) ① FLAIR 0.0012; 30.707; 0.9130; 0.0283T1 0.0017; 31.514; 0.9548; 0.0155 T1CE 0.0008; 32.631; 0.9311; 0.0265 T2 0.0010; 32.348; 0.9409; 0.0235	N/A	✕
2025/Guo, et.al [37]	Dataset from the Second Affiliated Hospital of Kunming Medical University/359 (115 benign, 244 malignant)/ultrasound images of lesions (B-mode), Color Doppler Flow Imaging (CDFI)	Proposed a multimodal liver tumor classification framework that uses generative models (U-GAT-IT, MSA-GAN) to reconstruct missing images for cross-modal supplementation	GAN	Annotation, histogram equalization.	Classification ① liver tumor	Accuracy (ACC); Precision (PRE); Recall ((REC); F1 score (F1S); AUC ① 88.57; 87.97; 86.32; 0.87; 0.95	N/A	✕
2024/Deng, et al. [38]	SYSU abdominal dataset/24 (1729 slices training, 299 slices testing/MRI, CT IXI/40 (30 training, 10 testing)/T1 MRI. T2 MRI (PD)	Integrating Channel Attention Module into the generator for better feature extraction, incorporating VGG16 and multiple loss terms	GAN, VGG16	Images registration, removal, normalization	① synthesized image quality measurement	PSNR; SSIM ① T1toT2 27.084; 0.875	N/A	√
2023/Zhang, Mengyi, et al. [39]	ADNI/370/MRI, FDG-PET	Synthesize PET images using a pyramidal attention mechanism and standard discriminators	GAN, U-net	-(MRI) spatial normalization, brain image segmentation, gray matter image acquisition, gray matter image modulation, image smoothing -(PET) head correction, registration and normalization, PET image normalization, PET image smoothing -(fusion) MRI brain gray matter segmentation, brain gray matter and metabolic information fusion Classification ① Alzheimer’s disease (AD) vs. Cognitively Normal (CN) ② Mild Cognitive Impairment (MCI) vs. CN ③ AD vs. MCI ④ AD vs. MCI vs. CN	Classification ① Alzheimer’s disease (AD) vs. Cognitively Normal (CN) ② Mild Cognitive Impairment (MCI) vs. CN ③ AD vs. MCI ④ AD vs. MCI vs. CN	ACC; Sensitivity (SEN); Specificity (SPE) ① missing PET 93.4; 97.5; 92.8 ② missing PET 92.5; 92.2; 92.5 ③ missing PET 93.1;91.5;97.3 ④ missing PET 89.9; 82.5; 85.9	N/A	✕
2023/Gao, Xingyu, et al. [40]	ADNI-1/821(196AD, 227CN,168pMCI, 230sMCI)/T1 MRI, T2 MRI, FDG-PET ADNI-2/534(156AD, 200CN,66pMCI, 112sMCI)/T1 MRI, T2 MRI, FDG-PET OASIS-3/345(174AD, 171CN)/T1 MRI, T2 MRI, FDG-PET	Use a feature autoregression branch and a task-level discriminator with transformer blocks; supervise local regions at voxel-level	GAN, U-net, transformer	Skull-stripping, region extraction, registration, transformation	Classification ① AD vs. CN ② progressive MCI (pMCI) vs. stable MCI (sMCI)	ACC; SEN; SPE; AUC ① missing T2MRI and PET (test on ADNI-2) 94.4; 93.0; 95.5; 97.6 (test on OASIS-3) 88.4; 84.6; 92.3; 94.4 ② missing T2MRI and PET (test on ADNI-2) 77.8; 75.4; 79.6; 82.8	N/A	✓
2023/Wang, Tonghui, et al. [41]	2Dslice of breast MRI/98(2245 slices) (65%training, 10%validation, 25%testing)/DCE-MRI, DWI	Utilize a GAN-like network to model the correlations between cross-sequence feature representations of different modalities	cycleGAN [98], ResNet	Manual and semi-automatic segmentation, lesion enhancement	Classification (breast tumor lesion task) ① slice level ② patient level	ACC; PRE; REC; F1S ① 0.8561; 0.8783; 0.8720; 0.8740 ② 0.8966; 0.9011; 0.9540; 0.9256	N/A	✕
2023/Jiang, Yang, Shuang Zhang, and Jianning Chi. [42]	BraTS 2018/285 (257 training, 28 testing)/MRI (T1, FLAIR, T1c and T2)	Train generator in a supervised manner with T2 images and multimodal classification labels, while a multi-branch convolutional neural network (CNN) is introduced as the discriminator	GAN, U-net	Filtering, resizing, labeling	① synthesized image quality measurement segmentation ② whole tumor (WT) ③ tumor Core (TC) ④ enhanced tumor (ET)	PSNR; SSIM ① T2toFLAIR 22.2560; 0.9478 T2toT1 29.9289; 0.9713 T2toT1c 24.6464; 0.9537 DSC ② 0.8836 ③ 0.8056 ④ 0.7366	N/A	✕
2023/Cao, Bing, et al. [43]	BraTS 2020/494 (369 training, 125 testing)/MRI (T1, FLAIR, T1c and T2) ISLES 2015/(28 training, 17 testing)/MRI (T1, FLAIR, DWI and T2) CBMFM/69 (62 training, 7 testing)/T1 MRI, CT	Transfer target-specific information to generator with multi-scale feature constraints and use an auto-encoder-based discriminator	GAN, auto-encoder	-(BraTS 2020 and ISLES 2015) resizing -(CBMFM) zero-padding, center-cropping	① synthesized image quality measurement	PSNR; SSIM; Feature similarity index measure (FSIM) ① (test on BraTS 2020) toT1 26.42; 93.42; 96.86 toT1c 29.45; 93.16; 96.94 toT2 26.91; 92.24; 96.34 toFLAIR 26.43; 90.44; 96.07 (test on ISLES 2015) toT1 24.24; 87.70; 94.47 toDWI 29.09; 91.35; 95.78 toT2 24.94; 85.33; 93.25 toFLAIR 29.08; 89.47; 95.07 (test on CBMFM) toCT 23.30; 83.34; 94.56	https://github.com/bcaosudo/AE-GAN.	✕
2023/Wang, Yulin, et al. [44]	IXI/578 (405 training, 58 validation, 115 testing)/T1 MRI. T2 MRI, (PD) BraTS 2021/1251 (500 training, 72 validation, 142 testing)/MRI (T1, FLAIR, T1CE and T2)	Utilize a hybrid generator and a CNN-based discriminator	CNN, transformer	-(IXI) spatial registration, cropping -(BraTS) Cropping -(both) Image blurring, down-sampling, interpolation	① random synthesis (SYN) ② super-resolution (SR) ③ segmentation	PSNR;SSIM ① (test on BraTS 2021) toT1CE 31.07; 0.949 (test on IXI) toPD 29.95; 0.945 ② (test on BraTS 2021) toT1CE 35.70; 0.983 (test on IXI) toPD 33.41; 0.975 DSC ③ (test on BraTS 2021) toT1CE 0.917	N/A	✕
2023/Liu, Jiang, et al. [45]	IXI/577 (521 training, 28 validation, 28 testing)/T1 MRI. T2 MRI, PD BraTS 2021/1251 (1123 training, 63 validation, 63 testing)/MRI (T1, FLAIR, DWI and T2)	Treat missing modality imputation as a sequence-to-sequence task, utilizing a transformer architecture that captures multi-contrast and multi-scale information	Transformer	-(IXI) Co-registration -(BraTS) Exclusion, cropping -(both) Normalization	① synthesized image quality measurement	PSNR; SSIM; LPIPS ① (test on BraTS 2021) missing one modal 27.87; 0.932; 0.104 (test on IXI) Missing one modal 36.31; 0.961; 0.080	N/A	✕
2023/Wu, Jianghao, et al. [46]	BraTS 2020/369 (258 training, 37 validation, 74 testing)/MRI (T1, FLAIR, DWI and T2)	Train and infer with cascaded dual-task architecture; use coarse segmentation to regularize the synthesis process and apply a tumor-informed loss function in discriminator to enhance synthesis quality	U-net	Cropping, normalization	① synthesized image quality measurement ② segmentation (WT)	Global SSIM; Local SSIM; Global PSNR; Local PSNR ① missing FLAIR 0.75; 0.52; 22.99; 20.01 DSC ② missing FLAIR 86.09	N/A	✕
2022/Jin, Leiming, et al. [47]	ADNI-1, ADNI2, ADNI-GO/360/MRI, PET	Use a pretrained classification network to guide a GAN for image synthesis	GAN, U-net, Resnet	Registration, skull stripping, segmentation, removal, resizing, normalization			N/A	✕
2022/Sun, Yuqing, Yong Liu, and Bing Liu. [48]	ADNI/363 (223 sCN, 9 pCN, 131 MCI)/T1 MRI, PET, clinical data	Reconstruct missing tau PET images from paired T1 MRI data	GAN	-(MRI) Segmentation, normalization -(PET) Co-registration, normalization -(all) Reslicing, normalization	Prediction ① CN vs. MCI	AUC; ACC; SEN; SPE; F1S ① missing PET 0.715; 0.713; 0.529; 0.848; 0.610	N/A	✕
2022/Zhang, Jin, et al. [49]	ADNI/873 (677 training, 31 validation, 165 testing)/T1 MRI, FDG-PET	Use 3D U-Net and generator with a 3D gradient profile loss and SSIM loss	GAN, U-net	Registration, alignment, removal, resizing, normalization	① synthesized image quality measurement	Mean absolute error (MAE); PSNR; SSIM ① missing PET, not splited by subjectID 0.0318; 26.92; 0.7294 Missing PET, splited by subjectID 0.0396; 25.08; 0.6646	N/A	✕
2022/Liu, Yunbi, et al. [50]	CLAS/76/T1 MRI ADNI-1, ADNI-2/1145/T1 MRI, FDG-PET AIBL/235/T1 MRI, Flutemetamol|Pittsburgh compound B (Flute|PIB)-PET	Combine two subnetworks, image synthesis and representation learning, sharing the same imaging features; use transfer learning to handle limited data problem	GAN	-(MRI) Skull-stripping, correction, normalization -(PET) Alignment, normalization	Classification ① progressive subjective cognitive decline (SCD) vs stable SCD ② pMCI vs. sMCI	AUC; Balanced accuracy (BAC); SPE; SEN; F1S ① missing PET (test on CLAS) 0.747; 0.721; 0.692; 0.750; 0.621 AUC; ACC ② missing PET (test on ADNI) 0.838; 0.780	https://github.com/Candyeeee/JSRL	✓
2022/Huang, Pu, et al. [51]	BraTS 2019/335 (80% training, 10% validation, 10% testing)/MRI (T1, FLAIR, T1c and T2)	Uses different encoders to separately process each type of medical image and extract important features and then use group convolution to combine these features from different image types into a single, unified representation	GAN, encoder–decoder	Alignment, normalization	① synthesized image quality measurement	PSNR; SSIM; Normalized mean squared error (NMSE) ① T1toT2 (whole image) 27.38; 0.953; 0.030 (tumor region) 18.08; 0.686; 0.007 T1toFLAIR (whole image) 28.41; 0.962; 0.014 (tumor region) 17.81; 0.607; 0.007	N/A	✕
2022/Kaplan, Sydney, et al. [52]	eLABE/127/T1 MRI, T2 MRI, rs-fMRI ECHO/10/T1 MRI, T2 MRI, rs-fMRI	Train 3D-GAN on full volumetric data following cycleGAN procedure	GAN	Distortion correction, denoising, normalization, registration, filtering	① synthesized image quality measurement	MAE; Mean structural similarity index measure (MSSIM); DSC ①T1toT2 0.056; 0.79; 0.82	N/A	✓
2021/Gao, Xingyu, et al. [53]	ADNI-1/821 (196 AD, 168 pMCI, 230 sMCI,227 CN)/T1 MRI, FDG-PET ADNI-2/534 (156 AD, 66 pMCI, 112 sMCI, 200 CN)/T1 MRI, FDG-PET	Combine pyramid convolution and attention module with a task-induced discriminator	GAN, U-net	Skull-stripping, registration, alignment, cropping, downsampling	① synthesized image quality measurement classification ② AD vs. CN ③ pMCI vs. sMCI	SSIM; PSNR; Mean squared error (MSE); Maximum mean discrepancy (MMD) ① missing PET 0.915; 29.0; 184; 0.107 ACC; SEN; SPE; AUC; F1S ② missing PET 92.0; 89.1; 94.0; 95.6; 90.5 ③ missing PET 75.3; 77.3; 74.1; 78.6; 69.9	N/A	✕
2021/Yan, Kun, et al [54]	BraTS2015/54 (42 training, 6 validation, 6 testing)/MRI (T1, FLAIR, T1c and T2)	Use a semi-supervised cross-modal MRI synthesis network based on cycleGAN adopting the coarse-to-fine learning strategy	GAN, denoising autoencoders [101]	Normalization, cropping	① synthesized image quality measurement	MSE; SSIM; PSNR ① (20%paired) T1toT2 0.0054; 0.9047; 23.85 T2toT1 0.0049; 0.9207; 23.79	N/A	✕
2021/Islam, Mobarakol, Navodini Wijethilake, and Hongliang Ren. [55]	BraTS 2017/331 (285 training, 46 testing)/MRI (T1, FLAIR, T1c and T2) TCGA/202/MRI (T1, FLAIR and T2), genomic data	Use a network that translates multi-modal MRI inputs into a single MRI modality, employing a fully convolutional network as the generator and an encoder–decoder architecture as the discriminator	GAN, FCN	Resampling, registration	① synthesized image quality measurement segmentation ② WT ③ TC ④ ET	PSNR ① T1,FLAIRtoT2 24.9387 DSC ② 0.8776 ③ 0.7615 ④ 0.7243	N/A	✓
2021/Akbar, Muhammad Usman, Vittorio Murino, and Diego Sona. [56]	Synapse/30 (24 training, 2 validation, 4 testing)/CT CHAOS/20 (16 training, 2 validation, 2 testing)/T1 MRI, T2 MRI\	Enhance the dataset by synthesizing one imaging modality from another modality sourced from a separate dataset	cycle-GAN	Re-sampling of CT, label balancing, normalization	Segmentation ① Liver ② Spleen ③ Right kidney ④ Left kidney	DSC ① CT 0.9519; MRI 0.9347 ② CT 0.9215; MRI 0.8463 ③ CT 0.8695; MRI 0.8948 ④ CT 0.8891; MRI 0.8330	N/A	✕
2021/Lin, Wanyun, et al. [57]	ADNI-1,2,3,GO/1086 (70% training, 20%validation, 10% testing)/MRI,PET	Use reversible structure and improve the generator part in GAN	3D GAN	-(MRI) Correction, resampling, cropping -(PET) Registration, cropping, normalization, averaging, resampling	Classification ① AD vs. CN ② pMCI vs. sMCI	ACC; SEN; SPE; AUC ① missing PET 89.05; 90.48; 87.50; 87.92 Missing MRI 88.64; 91.60; 85.71; 87.36 ② missing PET 71.23; 74.36; 67.65; 73.66 Missing MRI 71.18; 69.07; 73.97; 70.80	N/A	✕
2020/Pan, Yongsheng, et al. [58]	ADNI-1,2; the Australian Imaging/2355/MRI, PET	Invent a hybrid loss function containing three losses: voxel-wise-consistent loss, cycle-consistent loss, adversarial loss	GAN	N/A	① synthesized image quality measurement classification ② AD vs. CN ③ pMCI vs. sMCI	PSNR; SSIM; MAE ① toMRI 26.07; 0.6683; 0.1070 toPET 30.24; 0.6945; 0.0757 ACC; AUC; SEN; SPE; F1S; Matthews correlation coefficient (MCC) ② 93.58; 96.95; 91.52; 95.22; 92.64; 86.97 ③ 77.44; 82.51; 79.07; 77.22; 45.64; 40.06	N/A	✕
2020/Liu, Yunbi, et al. [59]	ADNI, CLAS/1055 (863 training (ADNI), 79 testing (ADNI), 113 testing (CLAS))/MRI, PET	Combine image synthesis network with a representation learning network; use transfer learning to handle limited data problem	3D-GAN	Skull-stripping, intensity correction, spatial normalization	Classification ① stable SCD vs. progressive SCD	AUC; ACC; SPE; SEN; F1S ① 0.713; 0.655; 0.616; 0.725; 0.598	N/A	✓
2020/Dar, Salman UH, et al. [60]	MIDAS/40 (25 training, 5 validation, 10 testing)/T1 MRI, T2 MRI IXI/40 (25 training, 5 validation, 10 testing)/T1 MRI, T2 MRI, PD BraTS2015/40 (25 training, 5 validation, 10 testing)/T1 MRI, T2 MRI, FLAIR Multi-Coil MRI/10 (7 training, 1 validation, 2 testing)/T1 MRI, T2 MRI, PD	Use two branches to jointly reconstruct and synthesize the target contrast	Conditional GAN [102]	Discarding, registration, upsampling	① synthesized image quality measurement	PSNR; SSIM ① (test on MIDAS) T1toT2 37.35; 97.96 T2toT1 32.17; 94.53 (test on IXI) T1toT2 36.18; 97.70 T2toT1 34.56; 97.91 (test on BraTS2015) T1toT2 36.15; 98.77 T2toT1 36.46; 98.86 (test on Multi-Coil) T1toT2 35.51; 97.61 T2toT1 36.40; 97.74	N/A	✕
2020/Cao, Bing, et al. [61]	ADNI/16/T1 MRI, CT BraTS/352 (80% training, 20% testing)/MRI (T1, FLAIR, T1c and T2)	Use auto-encoder network for self-supervised learning, and use a collaborative learning framework to utilize information from multiple modalities	GAN, auto-encoder	N/A	① synthesized image quality measurement	SSIM; FSIM ① missing T1 0.9317; 0.9657 Missing T1c 0.9061; 9.9573 Missing FLAIR 0.8948; 0.9602 Missing T2 0.9157; 0.9592	N/A	✕
2019/Sharma, Anmol, and Ghassan Hamarneh. [62]	ISLES2015/22 (training)/MRI (T1, T2, DWI, FLAIR) BraTS2018/285 (260 training, 10 validation, 15 testing)/MRI (T1,T2,T1C, FLAIR)	Use multi-input multi-output synthesizer using implicit conditioning and trained using curriculum learning	GAN, U-net	Mean normalization, cropping, resizing	① synthesized image quality measurement	MSE; PSNR; SSIM ① (mean) Missing T1 0.0052; 26.6057; 0.9276 Missing T2 0.0049; 26.1233; 0.9078	N/A	✕
2018/Pan, Yongsheng, et al. [63]	ADNI-1,2/821/MRI, PET	Impute missing modalities with cycle-consistent GAN to learn bi-directional mapping between different modalities	3D-cGAN	Alignment, skull stripping, correction, removal	Classification ① AD vs. healthy control (HC) ② pMCI vs. sMCI	ACC; SEN; SPE; F1S; MCC; AUC ① 92.50; 89.94; 94.53; 91.37; 84.78; 95.89 ② 79.08; 55.26; 82.85; 40.86; 30.13; 75.84	N/A	✕
2018/Tang, Zhenyu, Pew-Thian Yap, and Dinggang Shen. [64]	BraTS2015 100 (50 images out of 1000 for testing)/MRI (T1,T2,T1C, FLAIR) LPBA40/T1 MRI	Use synthesizers to generate multimodal normal atlases from standard single modality normal atlases	cycleGAN	Affine transformation, histogram matching	① segmentation	DSC (average) ① gray matter 0.701 White matter 0.748 CSF 0.593	N/A	✕

**Table 5:** Knowledge transfer methods dealing with missing imaging modality

Knowledge transfer methods								
Year/author/citation	Dataset/no. of subjects/modalities	Main context	Architecture	Preprocessing	Task	Metrics/performance	Code	External validation
2025/Liu, et al. [65]	BraTS2020, 2018, 2015/927 (660 training, 91 validation, 177 testing)/MRI (T1, FLAIR, T1c and T2)	Propose an enhanced model with two key stages: a pretraining phase synthesizing diverse MRI data by decoupling anatomy and tumor components, and a post-training phase applying knowledge distillation	3D encoder–decoder, 3D U-net	Background removal, normalization, cropping, augmentations	Segmentation ① whole tumor (WT) ② tumor core (TC) ③ enhanced tumor (ET)	Dice similarity coefficient (DSC) ① missing T1 (complete data) 91.15 (91.29) ② missing T1 and T2 (complete data) 86.21 (85.91) ③ missing T1 (complete data) 82.41 (82.09)	Code https://github.com/ZhongAobo/Asymmetry-BTS	**√**
2023/Xing, Xiaohan, et al. [66]	TCGA-GBM, TCGA-LGG project/737 (182 grade II, 205 grade III, 350 grade IV; 80%training, 20%testing)/ROI images, genomic features	Multi-modal teacher network trained for adaptive teaching of unimodal student network	ResNet	Augmentation (random cropping, color jittering, flipping)	① pathological glioma grading	Area under the curve(AUC); Average precision (AP); Accuracy (ACC); Kappa score ① missing 92.42, 86.34, 76.47, 64.34	https://github.com/CUHK-AIMGroup/MultiModal-learning.	✕
2023/Chen, Yuanyuan, et al. [67]	ADNI-1, ADNI-2, ADNI-GO/1248 (347 AD, 417 CN, 484 MCI; 80% training, 20% testing)/T1 sMRI, FDG PET	Use knowledge distillation model to impute representation of the missing modality	CNN	-(sMRI) Reorientation, normalization, registration, skull stripping, cerebellum removal -(PET) Smoothing, coregistration, averaging, computing AC-PC orient baseline, standardization, normalization -(both) Resizing, normalization	Classification ① Alzheimer’s disease (AD) vs. Cognitively Normal (CN) ② progressive MCI (pMCI) vs. stable MCI (sMCI)	AUC; AP; Sensitivity (SEN); Specificity (SPE); Matthews correlation coefficient (MCC) ① missing 96.85; 90.23; 91.73; 93.69; 84.21 ② missing 83.81; 77.26; 72.97; 79.02; 51.27	N/A	✕
2023/Dolci, G., et al. [68]	ADNI/1581 (644CN,332AD, 316MCI non-converters (MCInc), 289MCI converters (MCIc); 80% training, 20% testing)/sMRI, fMRI, Single nucleotide polymorphisms (SNP)	Recover missing modalities using pre-trained generators in the latent space	cycleGAN	-(sMRI) normalization, smoothing -(fMRI) rigid body motion correction, slice-timing correction, warping, resampling -(genomics data) genotyped, Pre-imputation QC, imputation	Classification ① AD vs. CN ② MCInc vs. MCIc	ACC; Precision (PRE); Recall ((REC) ① missing 0.938; 0.905; 0.884 ② missing 0.716; 0.622; 0.730	N/A	✕
2022/Dolci, Giorgio, et al. [69]	ADNI/788 (80% training and validation, 20% testing)/sMRI, fMRI, SNP	Use two cycleGAN to perform knowledge transfer from available modalities	cycleGAN	-(fMRI) correction, warping, resampling, smoothing -(sMRI) segmentation, smoothing -(both MRI) removal -(SNP) pre-imputation QC, imputation	Classification ① AD vs. CN	ACC; REC; PRE ① 87% at least one missing modal 0.935; 0.894; 0.894	N/A	✕
2022/Jeong, Seung-wan, et al. [70]	BraTS2017/285/MRI (T1, FLAIR, T1c and T2)	Use adversarial learning to generate the missing latent features and create a shared representation with an attention-based fusion block	ResNet	Skull stripping, co-registration, resizing, normalization, augmentation (flipping, rotation)	① tumor classification	ACC; AUC; SEN; SPE ① missingT1 (complete data) 88.75 (90.91); missingT1 (complete data) 95.58 (96.34); missingT2,FLAIR (complete data) 92.12 (92.69); missingT1 (complete data) 83.53 (84.90)	N/A	✕
2022/Yang, Qiushi, et al. [71]	BraTS2018/285 (195 training, 90 validation)/MRI (T1, FLAIR, T1c and T2)	Use a method where two models learn together by sharing detailed tumor-region information, guided by how similar different regions are, to improve performance	U^2^-net [105]	Normalization, random cropping, augmentation (random flipping, random rotation, random intensity change)	Segmentation ① whole tumor (WT) ② tumor core (TC) ③ enhanced tumor (ET)	DSC ① missing T1 (complete data) 88.8 (88.8) ② missing T2 (complete data) 80.9 (80.1) ③ missing T1,FLAIR (complete data) 68.7 (68.4)	https://github.com/CityU-AIM-Group/D2Net	✕
2022/Li, Haoran, et al. [72]	BraTS2020/369 (220 training, 74 validation, 75 testing)/MRI (T1, FLAIR, T1c and T2)	Use a Bernoulli sampling process to create training inputs for the student network; use a supervised knowledge transfer loss between teacher and student model	3D U-net	Co-registration, interpolation, skull-stripping	Segmentation ① WT ② TC ③ ET	DSC ① missing T1 (complete data) 89.7 (90.2) ② missing T1 (complete data) 87.2 (87.0) ③ missing FLAIR (complete data) 81.2 (82.3)	N/A	✕
2021/Vadacchino, Saverio, et al. [73]	BraTS2019/460 (335 training, 125 validation)/MRI (T1, FLAIR, T1c and T2)	Use a hierarchical discriminator which distills the latent information to overcome the large domain shift problem	3D U-net	N/A	Segmentation ① WT ② TC ③ ET	DSC ① missing T1c 87.5 ② missing T1c 66.7 ③ missing T1c 39.8	https://github.com/SaverioVad/HAD_Net	✕
2021/Wang, Yixin, et al. [74]	BraTS2018/285/MRI (T1, FLAIR, T1c and T2)	Use co-training approach between network composed of multimodal and unimodal paths	U-net	Co-registration, interpolation	Segmentation ① WT ② TC ③ ET	DSC; Hausdorff distance (HD95) ① missing T2 (complete data) 88.96 (89.22); 6.93 (6.71) ② missing T2,FLAIR (complete data) 84.59 (85.18); 5.76 (5.94) ③ missing T1,T2 (complete data) 77.46 (77.06); 4.22 (5.09)	https://github.com/Wangyix-inxin/ACN	✕
2020/Hu, Minhao, et al. [75]	BraTS2018/285 (2/3 training, 1/3 validation)/MRI (T1, FLAIR, T1c and T2)	Use a generalized knowledge distillation network, by constraining the latent representation of a mono-modal ‘student’ to be similar to multi-modal ‘teacher’	U-net	Cropping, random flipping, normalization, up-sampling	Segmentation ① WT ② TC ③ ET	DSC (only T1c) ① 76.98 ② 81.45 ③ 71.67	N/A	✕
2019/Shen, Yan, and Mingchen Gao. [76]	BraTS2018/361 (80% training, 20% validation)/MRI (T1, FLAIR, T1c and T2)	Use a loss function to make the internal features from missing modalities resemble those from complete ones	U-net	Normalization, cropping	Segmentation ① WT ② TC ③ ET	DSC ① missing T2 (complete data) 0.893 (0.894) ② missing T1 (complete data) 0.778 (0.790) ③ missing T2 (complete data) 0.643 (0.653)	N/A	✕

**Table 6:** Latent feature space-based methods dealing with missing imaging modality

Latent feature space-based methods								
Year/author/citation	Dataset/no. of subjects/modalities	Main context	Architecture	Preprocessing	Task	Metrics/performance	Code	External validation
2023/Zhou, Tongxue. [77]	BraTS 2018/285/MRI (T1, FLAIR, T1c and T2)	Integrate information across modalities, leverage latent multimodal correlations, and perform feature extraction	U-net [108]	Co-registration, interpolation, skull-stripping, manual labeling of ground truth, resizing, bias field correction, normalization	Segmentation ① whole tumor (WT) ② tumor core (TC) ③ enhanced tumor (ET) ④ average of three regions	Dice similarity coefficient (DSC) ① missing T2 (complete data) 86.6 (86.5) ② missing T2 (complete data) 87.1 (87.0) ③ missing T2 (complete data) 78.8 (78.6) ④ missing T2 (complete data) 84.1 (84.1)	N/A	✕
2023/Wang, Tao, et al. [78]	ADNI-1,2,3, OASIS/1530 (1387+143) divided into10 subsets (8 training, 1 validation, 1 testing)/MRI, PET	Impute missing data from multiple views spanning different modalities and temporal stages, incorporate the adversarial learning into the imputation to make it closer to the real distribution	MinimalRNN [109]	Correction, skull stripping, registration, segmentation, labeling	① Mild Cognitive Impairment (MCI) conversion prediction ② imputation assessment	Accuracy (ACC); Area under the curve (AUC); Balanced accuracy (BAC) ① ADNI1,2 0.842; 0.860; 0.830 ADNI3 0.813; 0.845; 0.821 Mean absolute error (MAE); Root mean squared error (RMSE) ② ADNI1,2 missing MRI 0.322; 0.468 missing PET 0.415; 0.513 OASIS missing MRI 0.372; 0.519 missing PET 0.621; 0.738	https://github.com/Meiyan88/MCNET	✕
2023/Gao, Xingyu, et al. [79]	ADNI-1,ADNI-2/1364(482 incomplete)/MRI,PET	A small network is used to estimate important abstract features of the missing data type using regression	Transformer [110]	Skull-stripping, linear registration, downsampling	Classification ① Alzheimer’s disease (AD) vs. cognitively normal (CN) ② progressive MCI (pMCI) vs. stable MCI (sMCI)	AUC; ACC; Sensitivity (SEN); Specificity (SPE) ① missing PET 96.7; 92.4; 88.5; 95.5 ② missing PET 87.2; 77.8; 75.4; 79.6	N/A	✕
2023/ Wang, Tao, et al. [80]	ADNI-1, ADNI-2/3166/T1 MRI, fluorodeoxyglucose (FDG)-PET, SNP PPMI/960/T1 MRI, DTI, SNP	Genetic data encoded using synthesized vectors to integrate with imaging data representations	ResNet [111]	-(MRI) Anterior commissure posterior correction, image intensity inhomogeneity correction, skull stripping, registration, segmentation, ROIs labeling, GM tissue volume computation -(PET) Co-registration -(DTI) b-vector and b-value file generation, eddy correction, skull stripping, fractional anisotropy calculation, alignment, ROIs labeling, mean tissue density calculation -(SNP) Quality control, removal, selection	Classification ① AD vs. CN ② pMCI vs. sMCI ③ Parkinson’s disease vs. CN ④ Parkinson’s disease vs. scans without evidence for dopaminergic deficit	AUC; ACC; SEN; SPE; Balanced classification accuracy (BCA) ① missing 93.81, 96.98, 93.40, 92.16, 93.10 ② missing 76.68, 78.08, 76.40, 77.35, 76.88 ③ missing 79.96, 81.56, 77.27, 80.62, 78. 95 ④ missing 80.31, 82.51, 88.51, 79.04, 83.78	https://github.com/Meiyan88/DMAAN	✕
2023/Wang, Hu, et al. [81]	BraTS2018/351 (285 training, 66 evaluation)/MRI (T1, FLAIR, T1c and T2)	Fuse shared and specific features of modalities with linear projection	3D U-net [112]	N/A	Segmentation ① WT ② TC ③ ET	DSC ① missing T1 (complete data) 90.79 (90.88) ② missing T1 (complete data) 85.67 (85.75) ③ missing T1&FLAIR (complete data) 78.59 (78.47)	https://github.com/billhhh/ShaSpec/	✕
2023/Morar, Ulyana, et al. [82]	ADNI/1843 (90% training, 10% testing)/MRI, PET, cerebrospinal fluid (CSF), biochemical biomarkers	Use a Long short-term memory (LSTM) [113] regressor and a pretrained Neural Network Estimator to impute missing values	LSTM	Augmentation, stratifying	Prediction ① Mini-Mental State Examination score	Correlation; RMSE; Coefficient of Determination ① 90.27; 1.86;81.36	N/A	✕
2023/Hou, Wentai, et al. [83]	TCGA/2102 (60%training, 20%validation, 20%testing)/clinical records, genomic data	Generate hyperedge using transformer to learn multi-modal dependence	Hybrid GCN [114]	Normalization	Prediction ① cancer survival	Concordance index (CI) ① KIRC: 0.750 LIHC: 0.695 ESCA: 0.664 LUSC: 0.590 LUAD: 0.639 UCEC: 0.735	https://github.com/lin-lcx/HGCN	✕
2022/Zhang, Yao, et al. [84]	BraTS 2018/285/MRI (T1, FLAIR, T1c and T2)	Inter-modal transformer combining all the input embeddings, and auxiliary regularizers	Transformer, CNN	Co-registration, interpolation, (augmentation) random flipping, cropping, intensity shifts	Segmentation ① WT ② TC ③ ET	DSC ① missing T1 (complete data) 88.14 (89.64) ② missing FLAIR (complete data) 80.39 (85.78) ③ missing T1 (complete data) 75.67 (77.61)	https://github.com/YaoZhang93/mmFormer	✕
2022/Hayat, Nasir, Krzysztof J. Geras, and Farah E. Shamout. [85]	MIMIC-IV; MIMIC-CXR/(70%training, 10%validation, 20%testing)/X-ray, clinical time series data	Flexible fusion framework designed to function without dependence on modality-specific encoders	LSTM	Sampling, discretization, standardization	① phenotype classification ② in-hospital mortality prediction	AUC;AUPRC ① 0.768; 0.429 ② 0.874; 0.567	https://github.com/nyuad-cai/MedFuse	✕
2022/Zhou, Tongxue, et al. [86]	BraTS 2018/285 (80%training, 20%testing)/MRI (T1, FLAIR, T1c and T2)	Combine multiple information sources and focus on the missing features via generator	U-net	Cropping, resizing	Segmentation ① WT ② TC ③ ET	DSC ① missing T2 87.1 ② missing T2 86.6 ③ missing T2 78.2	N/A	✕
2021/Zhu, Yian, et al. [87]	BraTS 2018/-(80%training, 20%validation)/MRI (T1, FLAIR, T1c and T2)	A model that uses two residual paths and multiple variational autoencoders to reconstruct features by adding small differences to the original input	U-net	Extraction of 2D slices, random normalization, cropping, augmentation (random axis mirror flipping)	Segmentation ① WT ② TC ③ ET	DSC ① missing T1 (complete data) 87.98 (87.90) ② missing T1 (complete data) 78.20 (78.82) ③ missing T2 (complete data) 79.10 (79.19)	N/A	✕
2021/Zhu, Yian, et al. [88]	BraTS 2018/-(80%training, 20%validation)/MRI (T1, FLAIR, T1c and T2)	Apply one module to generate shared features via cascade operation and another module to fuse the real features with generated ones.	U-net	Extraction of 2D slices, random normalization, cropping, augmentation (random axis mirror flipping)	Segmentation ① WT ② TC ③ ET	DSC ① missing T1c (complete data) 87.6 (88.3) ② missing T2 (complete data) 78.2 (77.7) ③ missing T2 (complete data) 69.3 (68.5)	N/A	✕
2021/Zhou, Tongxue, et al. [89]	-BraTS 2018/285 training, 66 validation; -BraTS 2019/335 training, 125 validation/MRI (T1, FLAIR, T1c and T2)	Use a correlation model and fusion strategy based on attention mechanism	U-net	Co-registration, interpolation, skull-stripping, manual labeling of ground truth, cropping, resizing, distortion correction, normalization	Segmentation ① WT ② TC ③ ET	DSC ① missing T1 (complete data) 87.9 (88.2) ② missing T1 (complete data) 77.5 (78.6) ③ missing T2 (complete data) 68.4 (69.4)	N/A	✕
2021/Huang, Ruobing, et al. [90]	Private dataset A/1560 (1022 training,100 validation, 438 testing)/ultrasound (B-mode, shear wave elastography, strain elastography, and Doppler) Private dataset B/163/B-mode	Recovery blocks use existing data to rebuild important features of missing modalities at an abstract representation level	CNN	Cropping, resizing	Classification ① breast nodule diagnosis	ACC; SEN; SPE; Precision (PRE); F1 score (F1S) ① (datasetA) Missing Doppler 90.65; 91.57; 89.67; 90.66; 91.10\ (datasetB) Only B-mode 79.26; 62.59; 87.52; 72.28; 66.52	N/A	✓
2020/Hu, Dan, et al. [91]	UNC/UMN Baby Connectome Project/178/structural MRI (sMRI), functional (fMRI)	Latent variable disentanglement strategy and imputation algorithm	CNN with adversarial autoencoder [115]	Co-registration, intensity inhomogeneity correction, skull stripping, cerebellum removal, tissue segmentation, hemispheres separation, topological correction, inner/middle/outer surface reconstruction	① missing imaging modality imputation	① Mean relative absolute error (MRAE);RMSE missing: 0.95; 0.677	N/A	✕
2019/van Garderen, Karin, Marion Smits, and Stefan Klein. [92]	BraTS 2018/278 (80%training, 20%testing)/MRI (T1, FLAIR, T1c and T2)	Curriculum learning approach used for fusing information of different modalities	U-net	Skull-stripping, co-registration, resampling, normalization	Segmentation ① WT ② TC ③ ET	DSC ① missing T1 (complete data) 81 (83) ② missing T1 (complete data) 64 (71) ③ missing T2 (complete data) 63 (63)	N/A	✕
2019/Lau, Kenneth, Jonas Adler, and Jens Sjölund. [93]	BraTS 2018/285 (70%training, 30%validation)/MRI (T1, FLAIR, T1c and T2)	Model trained to handle missing data using information from the available modalities, by training with randomly dropped modalities	U-net	Bias field correction, normalization, split	Segmentation ① WT ② TC ③ ET	DSC ① missing T1 (complete data) 86.0 (86.1) ② missing T1 (complete data) 78.8 (78.0) ③ missing T2 (complete data) 72.3 (71.3)	N/A	✕
2019/Dorent, Reuben, et al. [94]	BraTS 2018/285 (70%training, 10%validation, 20% testing)/MRI (T1, FLAIR, DWI and T2)	Extend Multi-modal Variational Auto-Encoders for 3D segmentation as well as mixture sampling strategy	3D U-net	Standardization (augmentation) random flipping, rotation	Segmentation ① WT ② TC ③ ET	DSC ① missing T1 (complete data) 88.6 (88.8) ② missing T1 (complete data) 75.6 (76.4) ③ missing T1 (complete data) 71.2 (71.7)	N/A	✕
2017/Chartsias, Agisilaos, et al. [95]	BraTS 2015/54 (42 training, 6 validation, 6 testing)/MRI (T1, FLAIR, DWI and T2) ISLES 2015/28 (22 training, 3 validation, 3 testing)/MRI (T1, FLAIR, T1c and T2) IXI/28 (22 training, 3 validation, 3 testing)/MRI (T1, T2, PD)	Model maps all input modalities to a common representation and uses one decoder per output type, guided by a custom loss to ensure consistency across modalities	Fully convolutional network (FCN) [116]	Trimming, normalization	① synthesized image quality measurement	Mean squared error (MSE); Structural similarity index measure (SSIM); Peak signal-to-noise ratio (PSNR) ① test on ISLES T1toT2 0.299; 0.831; 25.78 T1toFLAIR 0.268; 0.831; 29.99 test on BraTS T1toT2 0.333; 0.929; 30.96 T1toFLAIR 0.283; 0.897; 30.32	https://github.com/agis85/multimodal_brain_synthesis	✕
2016/Havaei, Mohammad, et al. [96]	MSGC/43 (20 training, 23 testing)/MRI (T1, FLAIR and T2) RRMS/300/MRI (T1, FLAIR, T1c and T2) BraTS2015/274/MRI (T1, FLAIR, T1c and T2) BraTS2013/35/MRI (T1, FLAIR, T1c and T2)	Learn a latent space embedding for each modality, enabling arithmetic operations within that space	CNN	Bias field correction, intensity normalization, truncation, unit variance, co-registration, interpolation	Segmentation ① WT ② TC ③ ET ④ lesion	DSC ① (BraTS) missing T1c (complete data) 83.87 (83.15) ② (BraTS) missing T1 (complete data) 70.62 (72.5) ③ (BraTS) missing T2 (complete data) 71.30 (75.37) ④ (RRMS) missing T1c (complete data) 46.6 (48.66)	N/A	✓

**Table 7:** Evaluation metrics used for evaluation of methods dealing with missing modalities

Evaluation metrics		
Metric	Formulation	Usage
Accuracy (ACC)	Given true positive (TP), true negative (TN), positive (P) and negative (N), $\text{ACC}=\frac{\text{TP}+\text{TN}}{\text{P}+\text{N}}$	ACC ranges from 0 to 1 (or in percentage terms), with the larger value showing that the classification result is more precise.
Sensitivity (SEN)/Recall ((REC)	Given true positive (TP) and positive (P), $\text{SEN}=\frac{\text{TP}}{\text{P}}$	SEN ranges from 0 to 1, with the larger value showing better performance in correctly identifying positive cases
Specificity (SPE)	Given true negative (TN) and negative (N), $\text{SPE}=\frac{\text{TN}}{\text{N}}$	SPE ranges from 0 to 1, with the larger value showing better performance in correctly identifying negative cases
Area under the curve (AUC)	$\text{AUC}=\int_{\text{x}=0}^{1}\text{TPR}\left({\text{FPR}}^{-1}(\text{x})\right)\text{dx}$ where FPR(T): T → x, the x-axis of ROC curve, denotes false positive rate, TPR(T): T → y(x), the y-axis of ROC curve, denotes true positive rate.	AUC ranges from 0.5 to 1, with the larger value showing that the model is more likely to arrange positive instance in front of negative instance (i.e. has a better ability of prediction).
Precision (PRE)	Given true positive (TP) and false positive (FP), $\text{Precision}=\frac{\text{TP}}{\text{TP}+\text{FP}}$	Higher precision values indicate better model performance in correctly identifying positive instances.
Average precision (AP)	$\text{AP}=\frac{\sum_{k=1}^{n}(P(k)\cdot \Delta R(k))}{\sum_{k=1}^{n}\delta (k)}$ where *n* is the total number of retrieved items, *P*(*k*) is the precision at cut-off k, Δ*R*(*k*) represents the change in recall from the previous cut-off to the current cut-off, *δ*(*k*) is an indicator function that is 1 if the item at position k is relevant, and 0 otherwise.	Higher AP values indicate better model performance, with 1 representing perfect precision and recall, and 0 representing no relevant items retrieved.
F1 score (F1S)	Given Precision (PRE) and Recall (REC), $F1=\frac{2\times \text{PRE}\times \text{REC}}{\text{PRE}+\text{REC}}$	F1 Score is widely used in binary classification tasks, especially when the classes are imbalanced. It ranges from 0 to 1, where 0 indicates poor performance, suggesting either low precision or low recall, 1 indicates perfect precision and recall.
Dice similarity coefficient (DSC)	Given two sets A and B $\text{DSC}=\frac{2\times |A\cap B|}{\left|A|+|B\right|}$	DSC is commonly used image segmentation, where the goal is to compare the similarity between two sets of regions. Higher Dice coefficient values indicate better agreement between the segmented regions and the ground truth, indicating better segmentation performance.
Balanced accuracy (BAC)	Given true positive (TP), false positive (FP), true negative (TN) and false negative (FN), $\text{BAC}=\frac{1}{2}\left(\frac{\text{TP}}{TP+FN}+\frac{TN}{TN+FP}\right)$	BAC is used to evaluate the performance of binary classification models, particularly with imbalanced datasets. Higher BAC values indicate better model performance, with 1 representing perfect classification accuracy and 0.5 representing random classification.
Balanced classification accuracy (BCA)	Given Sensitivity (SEN) and Specificity (SPE) $\text{BCA}=\frac{1}{2}(\text{SEN}+\text{SPE})$	BCA is also used to evaluate the performance of binary classification models, particularly with imbalanced datasets. Higher BCA values indicate better model performance, with 1 representing perfect classification accuracy and 0 representing random classification.
Matthews correlation coefficient (MCC)	Given true positive (TP), false positive (FP), true negative (TN) and false negative (FN), $\text{MCC}=\frac{TP\times TN-FP\times FN}{\sqrt{\left(TP+FP)(TP+FN)(TN+FP)(TN+FN\right)}}$	Higher MCC values indicate better classifier performance, with 1 representing perfect agreement between prediction and observation, 0 indicating random prediction, and −1 indicating total disagreement.
Mean absolute error (MAE)	$$\text{MAE}=\frac{1}{n}\sum_{i=1}^{n}\left|y_{i}-\hat{y_{i}}\right|$$ where n is the number of samples, ${\overset{ˆ}{y}}_{i}$ is sample I’s predicted value, and *y*_*i*_ is sample I’s true value	MAE is widely used as a measure of the accuracy of regression models. Lower MAE values indicate better model performance, as they suggest smaller errors between predictions and actual values.
Mean relative absolute error (MRAE)	$$\text{MRAE}=\frac{1}{n}\sum_{i=1}^{n}\left|\frac{y_{i}-\hat{y_{i}}}{y_{i}}\right|$$ where *n* is the number of samples, ${\overset{ˆ}{y}}_{i}$ is sample I’s predicted value, and *y*_*i*_, is sample I’s true value	In [91], MRAE is the mean of the absolute error divided by the corresponding chronological age. A lower MRAE indicates better model performance.
Mean squared error (MSE)	$$\text{MSE}=\frac{1}{n}\sum_{i=1}^{n}{\left(y_{i}-{\hat{y}}_{i}\right)}^{2}$$ where *n* is the number of samples, ${\overset{ˆ}{y}}_{i}$ is sample I’s predicted value, and *y*_*i*_ is sample I’s true value	MSE is commonly used for model evaluation, model comparison, and model selection in regression tasks. Lower MSE values indicate better model performance, as they suggest smaller errors between predictions and actual values.
Normalized mean squared error (NMSE)	$$\text{NMSE}=\frac{1}{n}\sum_{i=1}^{n}\frac{{\left(y_{i}-{\hat{y}}_{i}\right)}^{2}}{{\left(y_{i}-\bar{y}\right)}^{2}}$$ where n is the number of samples, ${\overset{ˆ}{y}}_{i}$ is sample I’s predicted value, *y*_*i*_ is sample I’s true value and $\bar{y}$ is the mean of true values.	In [51], NMSE was used to measure the model’s ability of image synthesis. A lower NMSE value indicates a better synthesis quality.
Root mean squared error (RMSE)	$$\text{RMSE}=\sqrt{\frac{1}{n}\sum_{i=1}^{n}{\left(y_{i}-{\hat{y}}_{i}\right)}^{2}}$$ where n is the number of samples, ${\overset{ˆ}{y}}_{i}$ is sample I’s predicted value, and *y*_*i*_ is sample I’s true value	In [78], RMSE was used to measure model’s ability of imputation. A smaller RMSE value indicates a better imputation performance.
Peak signal-to-noise ratio (PSNR)	Given the maximum possible pixel value (MAX) and Mean squared error (MSE) between the ground truth and synthesized images, $\text{PSNR}=10\cdot {\text{log}}_{10}\left(\frac{{\text{MAX}}^{2}}{\text{MSE}}\right)$	PSNR usually ranges from 20 to 50 (dB), with a larger value showing that the synthesized (reconstructed) image’s quality is better.
Structural similarity index measure (SSIM)	$$\text{SSIM}(x,y)=\frac{\left(2\mu_{x}\mu_{y}+C_{1})(2\sigma_{xy}+C_{2}\right)}{\text{(}\mu_{x}^{2}\text{+}\mu_{y}^{2}{\text{+C}}_{\text{1}}\text{)(}\sigma_{x}^{2}\text{+}\sigma_{y}^{2}+C_{2}\text{)}}$$ where C_1_ and *C*_2_ are constants to stabilize the division with weak denominator, μ_*x*_ is the mean of image *x*, $\sigma_{x}^{2}$ is the standard deviations of image x, σ_*xy*_ is the covariance of *x* and *y*	SSIM value is a decimal between −1 and 1, where 1 indicates perfect similarity, 0 indicates no similarity, and −1 indicates perfect dissimilarity. A higher SSIM value suggests a higher similarity between the images.
Mean structural similarity index measure (MSSIM)	Given Structural similarity index measure (SSIM), $\text{MSSIM}(x,y)=\frac{1}{mn}\sum_{i=1}^{m}\sum_{j=1}^{n}\;\text{SSIM}(x_{ij},y_{ij})$ where x and y are the compared images, m and n are the dimensions of the images, and x_ij, y_ijare the corresponding patches in images x and y.	In [52], a higher MSSIM value indicates higher similarity between images.
Learned perceptual image patch similarity (LPIPS) [117]	LPIPS(x,y)=1N∑i=1N1C∑c=1Cfc(xi)+fc(yi)R212 where *x* and *y* are two images, *N* is the number of patches, *C* is the number of channels, *f*_*c*_ is the feature extraction function, *R* is scaling factor.	In [45], LPIPS was utilized to capture the perceptual similarity between images. A lower LPIPS value indicates improved perceptual quality.
Feature similarity index measure (FSIM) [118]	$\text{FSIM}(x,y)=\frac{\sum_{i,j}S(x_{i},y_{i})\cdot f(x_{i},y_{i})}{\sum_{i,j}S(x_{i},y_{i})}$ where *x* and *y* are two images, *s* and f represents the local similarity and the structural feature similarity between corresponding image patches *x*_*i*_,*y*_*i*_ respectively.	In [43], a higher FSIM value indicates higher similarity between the compared images.
Maximum mean discrepancy (MMD)	${\text{MMD}}^{2}(ℱ,𝒢)={\left|\frac{1}{n(n-1)}\sum_{i=1}^{n}\sum_{j=1}^{n}\phi (x_{i})-\frac{2}{mn}\sum_{i=1}^{n}\sum_{j=1}^{m}\phi (x_{i})+\frac{1}{m(m-1)}\sum_{i=1}^{m}\sum_{j=1}^{m}\phi (y_{i})\right|}^{2}$ where *ϕ* represents for feature map, *x*_*i*_ and *y*_*i*_ are from distribution ℱ and 𝒢 respectively, while n and m are numbers of subjects of distribution ℱ and 𝒢 respectively.	In [53], a lower MMD value indicates better image quality.
Concordance index (CI)	$$CI=\frac{1}{N(N-1)}\sum_{i=1}^{N}\sum_{j=1}^{N}I\left(\hat{y_{l}}>\hat{y_{J}}\right)\cdot \delta \left(y_{i}<y_{j}\right)$$ where *N* is the total number of samples, $\hat{y_{l}}$ is sample I’s predicted value, *y*_*i*_ is sample I’s true value. A pair of *I* and *J* is concordant if the predicted ranking agrees with the true ranking (i.e., if $\hat{y_{l}}>\hat{y_{J}}$ and *y*_*i*_ < *y*_*j*_ and discordant otherwise.	In [83], CI was used to measure how well the model can reliably rank patients based on their predicted survival times. A larger CI, ranging from 0 to 1, indicates better performance.
Hausdorff distance (HD95)	Let *A* and *B* be two sets of points in a metric space, HD95(*A*, *B*) = max(percentile(*d*(*a*, *B*), 95),percentile (*d*(*b*, *A*), 95)) where *d*(*x*, *Y*) represents the distance between point *x* from set *X* and the nearest point in set *Y*, the function percentile(*x*,*p*) returns the pth percentile of the distances and the 95th percentile is chosen to calculate the maximum distance.	In [74], HD 95 was used to evaluate each nested subregion of brain tumors. A lower HD95 indicate better segmentation performance.

**Table 8:** Quantitative summary of the three methodological categories. Columns indicate (I) typical tasks, (II) median performance (DSC for segmentation, AUC for classification, SSIM for synthesis), (III) robustness to missing modalities, (IV) use of external validation, and (V) code availability

Category	Typical tasks	Median reported performance	Robustness	External validation	Code availability
Image synthesis methods	Mostly synthesis; segmentation; classification	DSC: ~0.86 (Segmentation) AUC: ~0.85 (Classification) SSIM: ~0.93 (Synthesis)	Limited (mainly tested for specific missing modality)	More frequently	Low (~10%)
Knowledge transfer methods	Mostly segmentation; classification	DSC: ~0.88 (Segmentation) AUC: ~0.84 (Classification)	Moderate (can handle missing modalities)	Rarely	High (~70%)
Latent space-based methods	Mostly segmentation; classification	DSC: ~0.86 (Segmentation) AUC: ~0.83 (Classification)	Moderate (can handle missing modalities)	Rarely	Moderate (~30%)

## Data Availability

Data sharing is not applicable to this article as no new data were created or analyzed in this study.
